# Artemotil Attenuates Fibrosis‐Associated Chondrocyte Remodelling in Osteoarthritis in Association With STC1‐Related TGF‐β/SMAD2/3 Signalling

**DOI:** 10.1111/jcmm.71374

**Published:** 2026-09-16

**Authors:** Guang Zhu, Yang Yang, Ye Ma, Gang Wu, Kuanmin Tian, Xiaoxin He, Rui Wang, Penggang Ma, Zhiqun Tang, Qunhua Jin

**Affiliations:** ^1^ The Third Ward of Orthopaedic Department, Institute of Osteoarthropathy, Institute of Medical Sciences General Hospital of Ningxia Medical University Yinchuan Ningxia Hui Autonomous Region People's Republic of China; ^2^ The First Clinical Medical College Ningxia Medical University Yinchuan Ningxia Hui Autonomous Region People's Republic of China

**Keywords:** Artemotil, cartilage fibrosis, osteoarthritis, STC1, TGF‐β/SMAD2/3 signalling

## Abstract

Cartilage fibrosis contributes to extracellular matrix remodelling and structural deterioration during osteoarthritis (OA) progression; however, the molecular regulators involved in this process remain incompletely understood. Stanniocalcin‐1 (STC1) has been implicated in tissue remodelling and fibrosis‐associated signalling, but its role in chondrocyte fibrotic phenotype transition remains unclear. In this study, primary mouse chondrocytes stimulated with interleukin‐1β (IL‐1β), a destabilization of the medial meniscus (DMM) mouse model, and human OA cartilage samples were used to investigate the involvement of STC1 in OA‐associated fibrotic remodelling. Quantitative proteomic profiling identified STC1 as a differentially regulated candidate associated with inflammatory and fibrotic responses. STC1 expression was elevated under OA‐related conditions and was accompanied by increased SMAD2/3 phosphorylation. In SW1353 chondrosarcoma‐derived chondrocytic cells, STC1 overexpression enhanced fibrosis‐associated phenotypic changes, including increased COL1A1, ADAMTS5, and MMP‐3 expression, reduced COL2A1 expression, and increased SMAD2/3 phosphorylation. Pharmacological modulation experiments further supported an association between STC1 and TGF‐β/SMAD2/3 signalling activity. Artemotil attenuated fibrotic and catabolic responses in IL‐1β‐stimulated chondrocytes, reduced cartilage degeneration in DMM‐induced OA, and was associated with reduced STC1 expression and reduced SMAD2/3 phosphorylation. Restoration of STC1 expression partially attenuated the effects of Artemotil on fibrosis‐related markers and signalling alterations. These findings support a functional association between STC1, TGF‐β‐responsive SMAD2/3 signalling, and fibrosis‐associated chondrocyte remodelling in OA and identify this signalling relationship as responsive to Artemotil intervention.

**Abbreviations**
ADAMTS: a disintegrin and metalloproteinase with thrombospondin motifs
COL1A1: collagen type I alpha 1 chain
COL2A1: collagen type II alpha 1 chain
ECM: extracellular matrix
IL‐1β: interleukin 1 beta
MMPs: matrix metalloproteinases
OA: osteoarthritis
SMAD2/3: mothers against decapentaplegic homologue 2/3
STC1: Stanniocalcin 1
TGF‐β: transforming growth factor beta

## 1 Introduction

Knee osteoarthritis (KOA) is a degenerative joint disorder characterized by progressive structural deterioration and extracellular matrix (ECM) remodelling [1, 2]. Chondrocyte phenotypic alterations and inflammation‐associated microenvironmental changes contribute to OA progression [3]. Despite extensive investigation, disease‐modifying pharmacological interventions for OA remain unavailable [4, 5, 6].

Increasing evidence suggests that cartilage fibrosis is involved in OA progression [7, 8]. This process is characterized by a transition from hyaline cartilage toward a fibrocartilage‐like phenotype associated with COL2A1 reduction and COL1A1 accumulation [9, 10]. This fibrotic phenotypic transition has emerged as an important pathological component of OA progression.

Artemisinin and its derivatives, originally developed as antimalarial agents [11], have attracted increasing attention due to their anti‐fibrotic and tissue‐remodelling regulatory properties in multiple pathological conditions, including renal, hepatic, and cardiac fibrosis [12, 13]. Artemisinin‐related compounds have been reported to regulate fibrosis‐associated signalling and extracellular matrix remodelling in several pathological contexts [14, 15, 16]. In OA‐related studies, artemisinin and its derivatives have been shown to affect cartilage degeneration, matrix‐degrading enzymes, mitochondrial autophagy, and mechanosensitive signalling [17, 18]. However, most studies have focused on inflammation and ECM degradation. The potential role of these compounds in cartilage fibrotic phenotypic alterations remains incompletely defined. Artemotil (β‐arteether; ART), a semisynthetic derivative with increased lipophilicity, may exhibit distinct biological properties compared with other artemisinin compounds. Previous studies have also linked artemisinin‐related compounds to TGF‐β‐associated signalling alterations and extracellular matrix remodelling in multiple pathological contexts [17, 19]. However, the effects of Artemotil on fibrotic phenotypic transition remain unclear.

Stanniocalcin‐1 (STC1) has emerged as a regulatory molecule involved in tissue remodelling, fibrosis‐associated signalling, and inflammatory responses [20, 21, 22]. Previous studies have demonstrated altered STC1 expression in OA‐related tissues [23, 24]; however, the findings remain inconsistent across different tissue types and experimental contexts. These observations indicate that STC1 may exhibit context‐dependent regulation in OA joints. In addition, bioinformatic and experimental studies have linked STC1 to signalling pathways associated with ECM remodelling and inflammatory responses, including TGF‐β‐related pathways [25, 26]. However, the role of STC1 in cartilage fibrosis and its relationship with chondrocyte phenotypic changes remain to be clarified.

In this study, we investigated whether STC1‐associated TGF‐β/SMAD2/3 signalling participates in fibrosis‐associated chondrocyte remodelling in OA and whether this axis is modulated by Artemotil treatment.

## 2 Materials and Methods

### 2.1 Animals

Male and female C57BL/6J mice, 10 weeks old, were obtained from the Laboratory Animal Center of Ningxia Medical University and housed at 21°C ± 1°C with 50% ± 5% relative humidity under a 12‐h light–dark cycle. The mice were provided standard chow and water ad libitum.

The mice were randomly assigned to experimental groups using a random number table (*n* = 6 per group). Male and female mice were distributed as evenly as possible across experimental groups to minimize potential imbalance in sex distribution. Because the study was designed to evaluate the overall effects of Artemotil rather than sex‐specific responses, data from male and female mice were pooled for the primary analyses. The sample size was not designed to provide adequate statistical power for sex‐stratified comparisons or sex‐by‐treatment interaction analyses. A formal a priori power analysis was not performed for sample‐size determination. All in vivo experiments and subsequent analyses were performed in a blinded manner to minimize potential bias.

All animal procedures were approved by the Ningxia Medical University Institutional Animal Care and Use Committee (IACUC‐H2025072) and were performed in accordance with the National Institutes of Health (NIH) guidelines for the care and use of laboratory animals.

### 2.2 Establishment of the OA Model

OA was induced using the destabilization of the medial meniscus (DMM) model, as previously described [27]. Mice were anaesthetized with isoflurane (2%–3% for induction and 1%–2% for maintenance), and all surgical procedures were performed under sterile conditions. A 3‐mm longitudinal incision was made from the distal patella to the proximal tibial plateau. The medial meniscotibial ligament was carefully transected to induce meniscal destabilization.

### 2.3 Experimental Groups and Drug Administration

The mice were randomly assigned into four groups: control (non‐operated), DMM + vehicle, DMM + ART (25 mg/kg; ART‐L), and DMM + ART (50 mg/kg; ART‐H) (*n* = 6 per group). ART doses of 25 and 50 mg/kg were selected with reference to dose ranges previously evaluated for arteether/β‐arteether in rodents [28, 29].

ART was administered via intraperitoneal injection once daily for 4 or 8 weeks starting on the day of surgery. Vehicle‐treated mice received an equivalent volume of the corresponding vehicle solution.

The mice were monitored throughout the treatment period, and no obvious signs of toxicity were observed. At the end of the experiment, knee joints were collected for histological analysis, and liver tissues were collected for safety evaluation. Independent cohorts of mice were used for each time point.

Humane endpoints were predefined for this study, including body weight loss exceeding 15%, impaired mobility, inability to access food or water, or signs of severe distress. Animals meeting these criteria were euthanized immediately. At the end of the experimental period, mice were euthanized by CO_2_ inhalation followed by cervical dislocation.

### 2.4 Primary Chondrocyte Isolation and Culture

Primary articular chondrocytes were isolated from the femoral condyles and tibial plateaus of 1‐week‐old C57BL/6J mice as part of the present study. Tissues were washed in PBS and digested overnight at 37°C with 1 mg/mL type II collagenase (MCE, HY‐E70005B). Cell suspensions were filtered and centrifuged at 100× *g* for 5 min. The cells were cultured in high‐glucose Dulbecco's Modified Eagle Medium (DMEM; Procell, PM150210) supplemented with 10% fetal bovine serum (FBS; Gibco, 10099141C) at 37°C with 5% CO_2_.

When cultures reached approximately 90% confluence, P0 cells were detached with 0.25% trypsin–EDTA (Gibco, 25200‐072) and replated. The chondrocytic identity and purity of the isolated primary cells were assessed before experimental treatment by double immunofluorescence staining for SOX9 and COL2A1, with DAPI used for nuclear counterstaining. The proportion of SOX9/COL2A1 double‐positive cells among total DAPI‐positive cells was quantified from three independent cell isolations. Only P0 or P1 cells were used for subsequent experiments.

P0 or P1 cells were treated with IL‐1β (10 ng/mL; PeproTech, 211‐11B) for 24 h to establish the in vitro inflammatory model. All experiments were performed with at least three independent biological replicates.

### 2.5 SW1353 Cell Culture and STC1 Overexpression

SW1353 chondrosarcoma‐derived chondrocytic cells (Anweisci, AW‐CH0340) were cultured in DMEM supplemented with 10% FBS at 37°C with 5% CO_2_. Because SW1353 cells are a chondrosarcoma‐derived cell line, they were used as an in vitro chondrocytic model for mechanistic manipulation, and key expression changes were further assessed in primary mouse chondrocytes and cartilage tissues.

### 2.6 Cell Viability Assay

Cell viability was assessed with a CCK‐8 kit (Servicebio, G4103‐1ML). Chondrocytes were plated into 96‐well plates (3 × 10^3^ cells/well) and treated with ART (0–100 μM) for 12, 24, 48, and 96 h. Absorbance at 450 nm was recorded using a Synergy HT microplate reader (BioTek). Cells treated with vehicle (0.1% DMSO) served as control.

### 2.7 Western Blotting

Cells were lysed using RIPA buffer (Beyotime, P0013B) supplemented with protease and phosphatase inhibitors. Protein concentrations were determined using a BCA protein assay kit (KeyGEN, KGB2101). Equal concentrations of protein (20 μg per lane) were separated by 10% SDS–PAGE and transferred onto PVDF membranes.

The membranes were blocked with 5% skim milk in TBST for 1 h at room temperature and then incubated overnight at 4°C with primary antibodies. After washing with TBST, the membranes were incubated with HRP‐conjugated secondary antibodies (ZSGB‐BIO) for 1 h at room temperature. Protein bands were visualized using enhanced chemiluminescence (BIOSHARP, BL520A) and quantified using ImageJ software.

Primary antibodies were used at the following dilutions: STC1 (Proteintech, 20621‐1‐AP, 1:1000), COL1A1 (Abcam, ab34710, 1:1000), COL2A1 (Bioss, bs‐10589R, 1:1000), MMP‐3 (Abcam, ab52915, 1:1000), MMP‐13 (Abcam, ab39012, 1:1000), ADAMTS5 (Bioss, bs‐3573R, 1:1000), ADAMTS4 (Bio‐Rad, BS‐4191R, 1:1000), Smad2/3 (ZenBio, 382472, 1:1000), p‐Smad2/3 (Proteintech, 80427‐2‐RR, 1:1000), Smad1/5 (ZenBio, 252529, 1:1000), and p‐Smad1/5 (Huabio, HA722566, 1:1000). β‐actin (Proteintech, 66009‐1‐Ig, 1:50,000) was used as a loading control.

### 2.8 Public Dataset Analysis (GSE75181)

The GEO dataset GSE75181 was downloaded for external validation of STC1 expression in OA cartilage, including 12 OA and 12 non‐OA samples.

Raw data were log2‐transformed and normalized using the limma package in R. Differential expression analysis was performed with limma, using the Benjamini–Hochberg correction for multiple testing. Genes with |log2 fold change| > 1.5 and adjusted *p* < 0.05 were considered differentially expressed.

### 2.9 Quantitative Proteomics

SW1353 cells were divided into three groups, each with four biological replicates (*n* = 4): control, IL‐1β, and IL‐1β plus ART. The experimental cells were treated with IL‐1β (10 ng/mL) for 24 h, with or without ART.

Protein extraction, digestion, and peptide preparation were performed as described above. Peptides were analysed using a NanoElute UHPLC system coupled with a timsTOF Pro mass spectrometer under dia‐PASEF acquisition mode. Data were processed using DIA‐NN software (version 1.8) with a false discovery rate of 1% at the protein level.

Differentially expressed proteins were defined using a fold change threshold > 1.5 or < 0.67 with *p* < 0.05. Proteins were ranked according to fold change and significance. STC1 was identified as a reproducibly altered protein across experimental conditions and was selected for further investigation based on its reported involvement in tissue remodelling and fibrosis‐related signalling.

### 2.10 Molecular Docking and Molecular Dynamics Simulation

Molecular docking and molecular dynamics simulations were performed to evaluate potential structural compatibility between ART and STC1. ART structures were prepared using ChemOffice, and protein structures were obtained from the AlphaFold database. Docking was performed with AutoDock Vina, and simulations were run with Gromacs 2022.

These analyses provided theoretical predictions of structural compatibility. They were not intended to establish direct binding interactions.

### 2.11 Human Cartilage Samples and Immunofluorescence

Articular cartilage samples were obtained from eight patients with primary knee OA undergoing total knee arthroplasty and were included for translational validation.

Patients aged 50–70 years who met the American College of Rheumatology (ACR) diagnostic criteria for primary OA were included. Patients with connective tissue diseases, a history of joint trauma, or secondary OA caused by metabolic disorders were excluded. Patients with systemic diseases that could affect joint pathology were also excluded.

During surgery, paired cartilage samples were collected from the weight‐bearing medial tibial plateau and the relatively preserved lateral tibial plateau of the same patients. Samples were then fixed, decalcified, and embedded in paraffin for subsequent analysis.

Immunofluorescence staining was performed to assess STC1 expression. All of the staining results were independently evaluated by two blinded investigators, and fluorescence intensity was quantified using ImageJ software.

This study was approved by the Medical Ethics Review Committee of General Hospital of Ningxia Medical University (approval no. KYLL‐2024‐153), and written informed consent was obtained from all participants.

### 2.12 Immunohistochemistry and Immunofluorescence

Cells or tissue sections were incubated with primary antibodies overnight at 4°C, followed by incubation with Alexa Fluor 488‐ or 594‐conjugated secondary antibodies (1:1000) for 1 h at room temperature. Nuclei were counterstained with DAPI. Images were captured using a Leica DMi8 fluorescence microscope. The primary antibodies used for immunofluorescence in the cultured chondrocytes and cartilage sections included STC1 (1:200), COL1A1 (1:200), COL2A1 (1:200), SOX9 (1:200), and p‐SMAD2/3 (1:200), unless otherwise specified.

For immunohistochemical analysis of p‐SMAD1/5, paraffin‐embedded knee joint sections were deparaffinized, rehydrated, and subjected to antigen retrieval. After blocking endogenous peroxidase activity and nonspecific binding, the sections were incubated with the p‐SMAD1/5 primary antibody (1:200) overnight at 4°C. Immunoreactivity was visualized using DAB, followed by haematoxylin counterstaining. The p‐SMAD1/5‐positive area within predefined articular cartilage regions was quantified using ImageJ.

For negative controls of tissue immunofluorescence staining, the primary antibody was replaced with the corresponding species‐matched non‐immune IgG, and the sections were subsequently processed using the same secondary antibody and imaging procedure. Quantification of cartilage fluorescence was restricted to predefined articular cartilage regions of interest.

All of the staining results were independently evaluated by two blinded investigators. Quantitative analysis was performed using ImageJ to assess fluorescence intensity.

### 2.13 Histology

Cartilage degeneration was evaluated using the Osteoarthritis Research Society International (OARSI) scoring system (0–6 scale) [30].

All histological evaluations were independently performed by two blinded investigators. Quantitative analysis was conducted using ImageJ software according to predefined criteria.

### 2.14 Quantitative Real‐Time PCR (RT–qPCR)

Total RNA was extracted from primary mouse chondrocytes using TRIzol reagent (Invitrogen, USA) according to the manufacturer's instructions. RNA concentration and purity were determined using a NanoDrop spectrophotometer (Thermo Fisher Scientific, USA). Complementary DNA (cDNA) was synthesized using a reverse transcription kit (Takara, Japan).

RT–qPCR was performed using SYBR Green Master Mix (Takara, Japan) on an ABI 7500 Real‐Time PCR System (Applied Biosystems, USA). The amplification conditions were set according to the manufacturer's protocol.

Relative gene expression levels were calculated using the 2^−ΔΔCt^ method and normalized to GAPDH expression. Primer sequences for STC1, COL1A1, and MMP3 are listed in Table S1.

All experiments were performed with at least three independent biological replicates.

### 2.15 Micro‐Computed Tomography (Micro‐CT) Analysis

Knee joint samples were scanned using a micro‐CT system (VNC‐102, PingSheng Medical Technology, China). Scanning parameters included a tube voltage of 90 kV, a tube current of 0.09 mA, and a voxel size of 0.026 mm.

Raw projection data were reconstructed using Recon software with a Feldkamp–Davis–Kress (FDK) algorithm. Three‐dimensional reconstruction and quantitative analysis were performed using Avatar software.

Bone microarchitecture parameters, including bone volume fraction (BV/TV), tissue mineral density (TMD), and trabecular separation (Tb.Sp), were quantified.

All analyses were performed in a blinded manner.

### 2.16 Statistical Analysis

All statistical analyses were performed using GraphPad Prism (version 10.0). The data are presented as the mean ± standard deviation (SD).

The normality of the data distribution was assessed using the Shapiro–Wilk test. For normally distributed data, one‐way ANOVA followed by the Bonferroni post hoc test was applied. For non‐normally distributed data, the Kruskal–Wallis test followed by Dunn's post hoc analysis was used.

For paired comparisons between medial and lateral tibial plateau cartilage from the same patients, paired Student's *t*‐test or Wilcoxon matched‐pairs signed‐rank test was used according to data distribution.

OARSI scores were analysed using the Kruskal–Wallis test followed by Dunn's post hoc test.

The statistical methods used for each experiment are indicated in the corresponding figure legends; *p* < 0.05 was considered to indicate significance.

## 3 Results

### 3.1 Artemotil Alleviates IL‐1β‐Induced Fibrotic and Catabolic Changes in Chondrocytes

**FIGURE 1:**
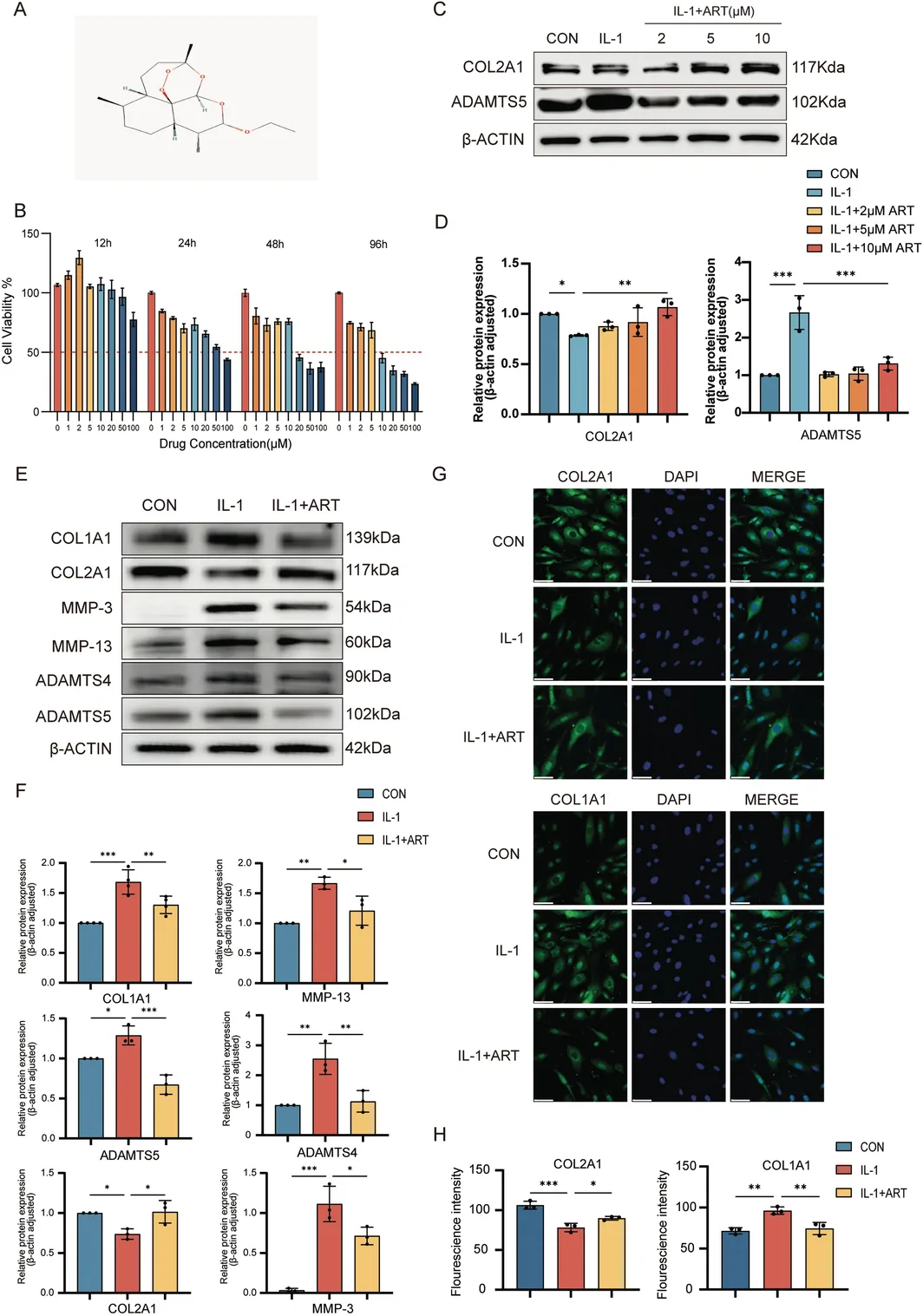
Artemotil suppresses fibrotic phenotypic transition in IL‐1β‐stimulated primary mouse chondrocytes. (A) Chemical structure of Artemotil (ART). (B) Cell viability of primary chondrocytes treated with ART (1–100 μM) for 12, 24, 48, and 96 h, assessed by CCK‐8 assay. (C, D) Western blotting of COL2A1 and ADAMTS5 expression in control, IL‐1β‐treated, and ART‐treated chondrocytes at different ART concentrations. (E, F) Western blotting of COL1A1, MMP‐3, MMP‐13, ADAMTS4, ADAMTS5, and COL2A1 expression under IL‐1β stimulation with ART treatment (10 μM). (G, H) Immunofluorescence staining and quantitative analysis of COL2A1 and COL1A1 in the control, IL‐1β, and IL‐1β + ART groups. Scale bar, 25 μm. The 10 μM concentration was selected for subsequent experiments according to dose–response observations. Data are presented as the mean ± SD, with individual data points shown (*n* = 3). Statistical analysis was performed using one‐way ANOVA followed by Bonferroni post hoc test. *p < 0.05, **p < 0.01, ***p < 0.001.

Primary chondrocytes isolated from mouse articular cartilage were used for the in vitro experiments. Double immunofluorescence staining showed nuclear SOX9 expression together with COL2A1 expression in the majority of isolated cells. Quantitative analysis showed that approximately 89% of DAPI‐positive cells were positive for both SOX9 and COL2A1, supporting the chondrocytic identity and high purity of the primary cultures used in subsequent experiments (Figure S1A,B). The chemical structure of ART is shown in Figure 1A [28]. Cell viability was assessed using a CCK‐8 assay following treatment with ART at different concentrations and time points. No apparent reduction in cell viability was observed within 24 h, whereas treatment with concentrations above 20 μM reduced viability at 48 h (Figure 1B).

Given these observations, a range of ART concentrations was further evaluated under inflammatory conditions. IL‐1β stimulation was used to establish an inflammatory chondrocyte model [31]. Western blot analysis showed that IL‐1β treatment reduced COL2A1 expression and increased ADAMTS5 expression compared with that in the control group (Figure 1C,D). With increasing ART concentrations, COL2A1 expression showed an upward trend, whereas ADAMTS5 expression showed a downward trend. Given these results, 10 μM was used for subsequent experiments.

Further analysis showed that IL‐1β increased COL1A1 expression and reduced COL2A1 expression, accompanied by increased expression of ADAMTS4, ADAMTS5, MMP‐3, and MMP‐13 (Figure 1E,F). Artemotil attenuated IL‐1β‐induced fibrotic and catabolic responses in chondrocytes.

RT–qPCR analysis showed that IL‐1β increased COL1A1 and MMP‐3 mRNA levels, whereas ART reduced their expression (Figure S1C).

Immunofluorescence analysis showed decreased COL2A1 and increased COL1A1 signals following IL‐1β stimulation, and this effect was partially reversed by ART (Figure 1G,H).

These findings indicate that Artemotil attenuates cartilage fibrosis–related phenotypic alterations under inflammatory conditions.

### 3.2 Fibrosis‐Associated Cartilage Degeneration Is Attenuated by Artemotil in DMM‐Induced Osteoarthritis

**FIGURE 2:**
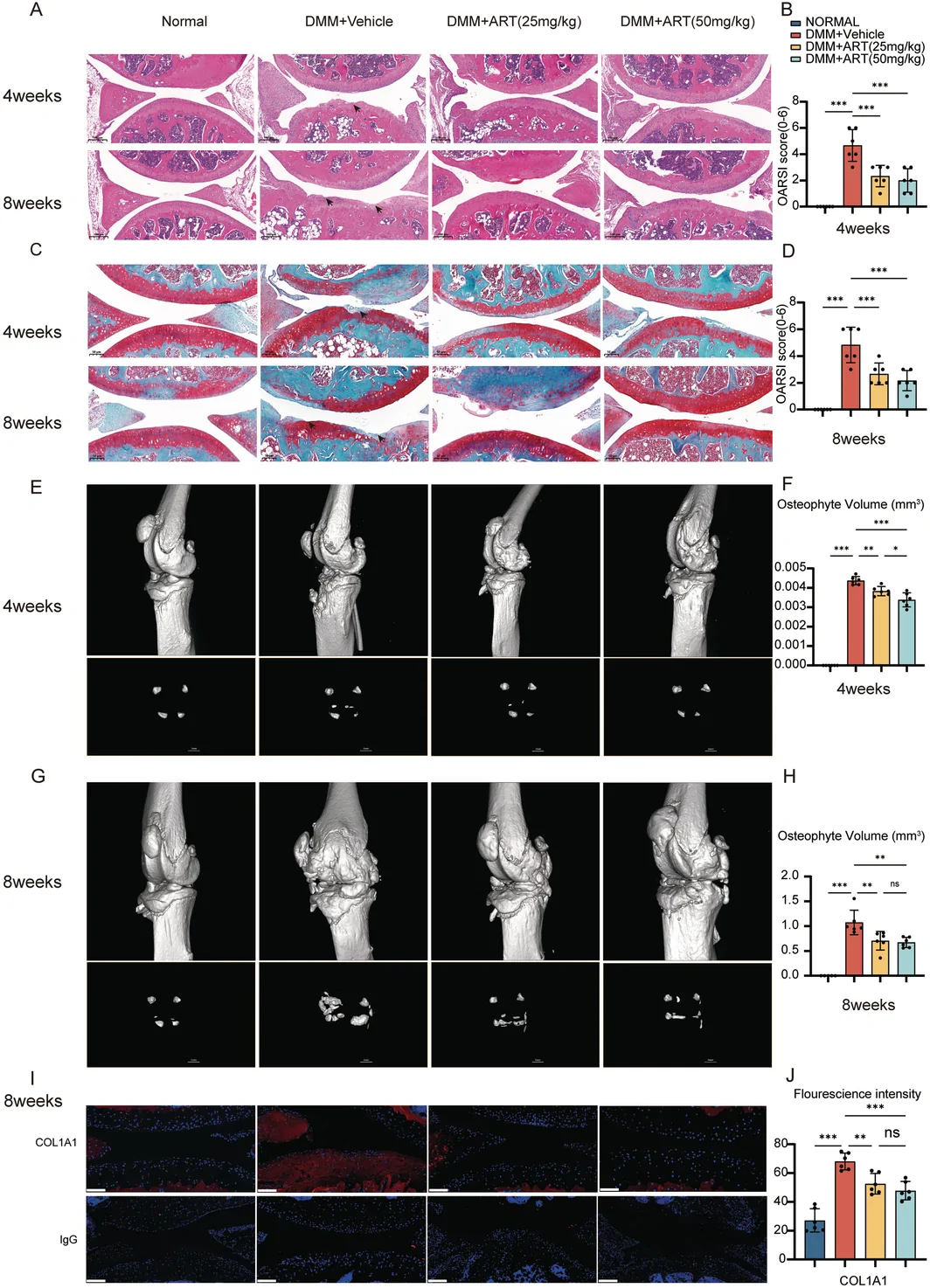
Artemotil attenuates OA‐associated cartilage destruction and osteophyte formation in a DMM‐induced osteoarthritis model. (A, C) Representative H&E and Safranin O–Fast Green staining of knee joint sections from the control, DMM + vehicle, DMM + Artemotil (ART) (25 mg/kg), and DMM + ART (50 mg/kg) groups at 4 and 8 weeks. (B, D) OARSI scores for quantitative evaluation of cartilage degeneration. (E–H) Micro‐CT images and quantitative analysis of osteophyte formation in each group at 4 and 8 weeks. (I) Representative immunofluorescence staining of COL1A1 and corresponding IgG negative controls in knee joint cartilage at 8 weeks. (J) Quantification of COL1A1 fluorescence intensity in the predefined articular cartilage region. Scale bars: Histological staining, 50 μm; immunofluorescence, 50 μm. Data are presented as the mean ± SD, with individual data points shown (*n* = 6 per group). OARSI scores were analysed using the Kruskal–Wallis test followed by Dunn's post hoc test. Other quantitative data were analysed using one‐way ANOVA followed by Bonferroni post hoc test. *p < 0.05, **p < 0.01, ***p < 0.001.

A DMM‐induced OA mouse model was used to evaluate the in vivo effects of ART. Mice were treated with ART (25 or 50 mg/kg) or vehicle for 4 or 8 weeks. Histological analysis using H&E and Safranin O–Fast Green staining showed cartilage surface disruption, structural irregularity, and reduced matrix staining in the DMM + Vehicle group (Figure 2A,C). In contrast, the ART‐treated groups exhibited improved cartilage morphology and preservation of matrix staining.

OARSI scoring further supported these observations, showing lower cartilage damage scores in ART‐treated mice compared with those in the DMM + Vehicle group at both time points (Figure 2B,D). A reduction in OARSI scores was observed in both treatment groups.

Micro‐CT analysis was performed to assess osteophyte formation. The DMM + Vehicle group showed pronounced osteophyte formation around joint margins; in contrast, ART‐treated mice exhibited reduced osteophyte size and number (Figure 2E–H). The quantitative analysis revealed decreased osteophyte volume in the ART‐treated groups at 4 and 8 weeks.

To further assess cartilage phenotypic alterations in vivo, we examined COL1A1 expression in knee joint cartilage. Immunofluorescence staining showed relatively low COL1A1 signals in control cartilage and increased staining in the DMM + Vehicle group. Reduced COL1A1 fluorescence intensity was observed in the ART‐treated groups (Figure 2I,J). Corresponding IgG controls showed only minimal background fluorescence without the tissue‐associated staining pattern observed with the COL1A1 antibody.

To further evaluate changes in the hyaline cartilage phenotype, COL2A1 expression was examined in articular cartilage at 8 weeks. COL2A1 fluorescence intensity was reduced in the DMM + Vehicle group compared with the control group, whereas higher COL2A1 signals were observed in the ART‐treated groups (Figure S1D,E). These findings further support the association of DMM‐induced OA with fibrosis‐related cartilage remodelling and loss of hyaline cartilage‐associated matrix characteristics. No obvious histological abnormalities were observed in liver tissues under the present experimental conditions (Figure S2).

These findings indicate that Artemotil attenuates OA‐associated cartilage degeneration, osteophyte formation, and fibrosis‐related remodelling in the DMM‐induced OA model.

### 3.3 Artemotil Attenuates Abnormal Subchondral Bone Remodelling in DMM‐Induced OA

**FIGURE 3:**
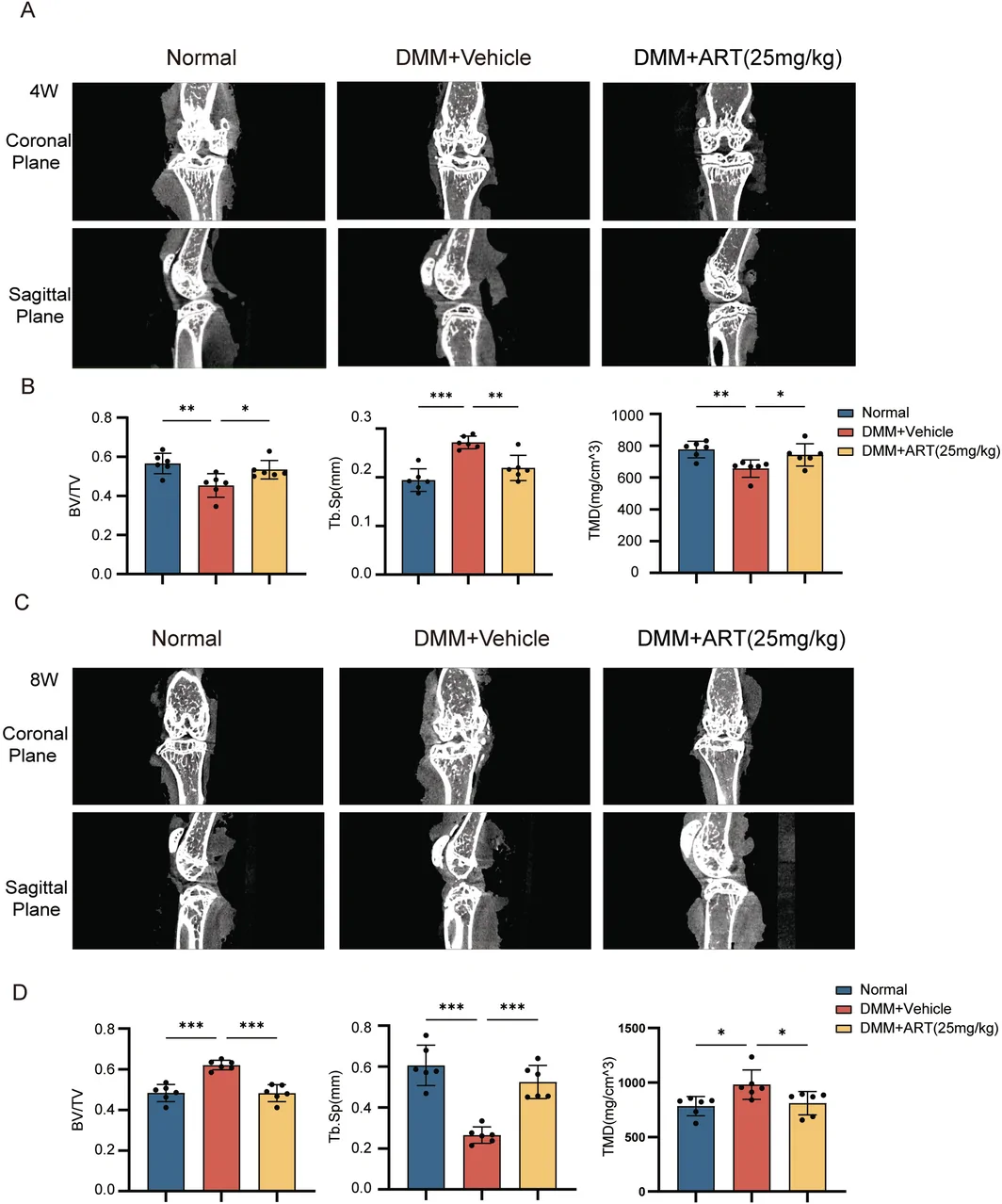
Artemotil attenuates aberrant subchondral bone remodelling in a DMM‐induced osteoarthritis model. (A) Representative micro‐CT images of knee joints in the coronal and sagittal planes at 4 weeks after DMM surgery in the control, DMM + vehicle, and DMM + Artemotil (25 mg/kg) groups. (B) Quantitative analysis of subchondral bone parameters at 4 weeks, including bone volume fraction (BV/TV), tissue mineral density (TMD), and trabecular separation (Tb.Sp). (C) Representative micro‐CT images at 8 weeks after DMM surgery. (D) Quantitative analysis of subchondral bone parameters at 8 weeks. Data are presented as the mean ± SD, with individual data points shown (*n* = 6 per group). Statistical analysis was performed using one‐way ANOVA followed by Bonferroni post hoc test. *p < 0.05, **p < 0.01, ***p < 0.001.

Micro‐CT analysis was performed to evaluate subchondral bone changes in the DMM‐induced OA model. At 4 weeks post‐surgery (Figure 3A,B), the control group exhibited intact subchondral bone architecture with regularly arranged trabeculae. In contrast, the DMM + Vehicle group exhibited disrupted microarchitecture, characterized by reduced BV/TV and TMD, along with increased Tb.Sp. ART treatment partially preserved subchondral bone structure, as indicated by improved trabecular organization. Quantitative analysis showed increased BV/TV compared with that in the DMM + Vehicle group.

At 8 weeks post‐surgery (Figure 3C,D), the DMM + Vehicle group showed alterations in subchondral bone microarchitecture, accompanied by increased BV/TV and TMD and reduced Tb.Sp. These features are consistent with sclerotic changes in subchondral bone. In contrast, ART‐treated mice exhibited attenuation of these alterations, with parameters closer to those observed in the control group.

Collectively, these data indicate that ART attenuates abnormal subchondral bone remodelling in the DMM‐induced OA model.

### 3.4 Proteomic Profiling Identifies STC1 as a Candidate Molecule Associated With Chondrocyte Fibrotic Remodelling

**FIGURE 4:**
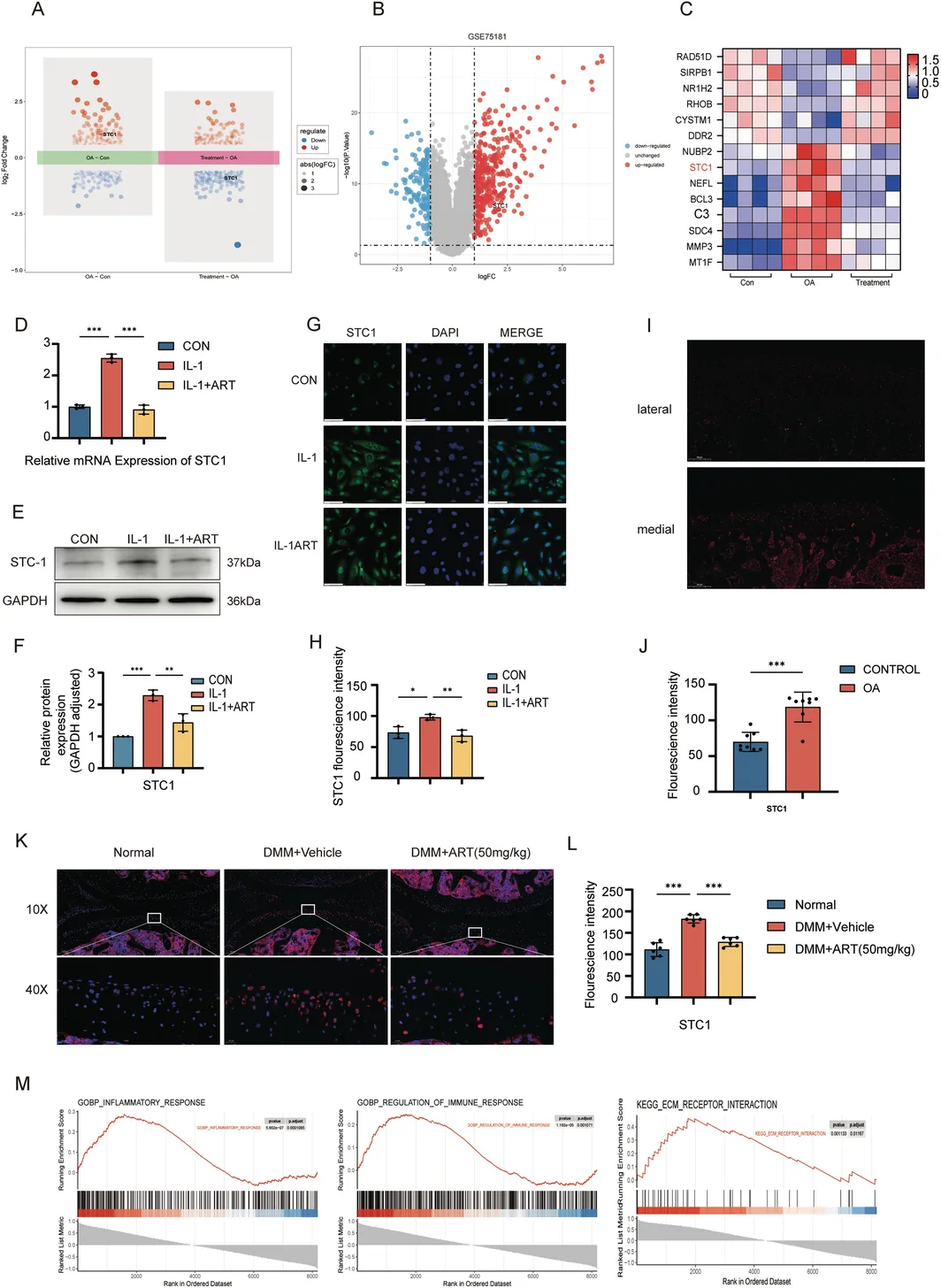
Proteomic identification of STC1 in IL‐1β‐stimulated SW1353 and validation in OA models. (A) Volcano plot showing differentially expressed proteins identified by quantitative proteomics in the control, IL‐1β‐stimulated, and Artemotil‐treated SW1353 cells. (B) Volcano plot of the GSE75181 dataset showing differential gene expression between OA and non‐OA cartilage, with STC1 indicated. (C) Heatmap of selected differentially expressed proteins across groups. (D) RT–qPCR analysis of STC1 mRNA expression in primary chondrocytes. (E, F) Western blotting and quantification of STC1 expression in primary chondrocytes. (G, H) Immunofluorescence staining and quantification of STC1 in primary chondrocytes. Scale bar, 25 μm. (I, J) Immunofluorescence staining and quantitative analysis of STC1 expression in paired human articular cartilage samples from the medial and lateral tibial plateaus of patients with OA (*n* = 8). (K, L) Immunofluorescence staining and quantification of STC1 in mouse articular cartilage. (M) Single‐gene GSEA of STC1 showing enrichment of biological processes. Quantitative data are presented as the mean ± SD with individual data points shown. Proteomic analysis was based on four biological replicates per group (*n* = 4). Primary chondrocyte experiments were performed with three independent biological replicates (*n* = 3), human cartilage comparisons included eight paired samples (*n* = 8), and in vivo experiments included six mice per group (*n* = 6). Differential expression analysis of GSE75181 was performed using limma as described in the Materials and Methods. Paired human cartilage comparisons were analysed using a paired Student's *t*‐test or Wilcoxon matched‐pairs signed‐rank test according to data distribution. Other quantitative comparisons were analysed as specified in the Materials and Methods. *p < 0.05, **p < 0.01, ***p < 0.001.

Quantitative proteomic analysis was performed in the control, IL‐1β‐stimulated, and ART‐treated cells, revealing differential protein expression across groups (Figure 4A,C). Differentially expressed proteins were defined according to fold change and statistical thresholds. Among these proteins, STC1 was identified as a regulated candidate, showing increased expression under IL‐1β stimulation and reduced expression following ART treatment. STC1 was prioritized for subsequent investigation because of its consistent regulation across inflammatory, experimental osteoarthritis, and pharmacological intervention conditions, as well as its reported involvement in tissue remodelling and fibrosis‐associated signalling.

We analysed the public dataset GSE75181 to further examine STC1 expression in human cartilage. Differential expression analysis using predefined criteria revealed that STC1 expression was upregulated in OA cartilage compared with non‐OA samples (Figure 4B).

To further validate these findings across different molecular levels, we conducted RT–qPCR analysis, revealing that IL‐1β stimulation increased STC1 mRNA levels, whereas ART treatment reduced its expression (Figure 4D).

Western blotting further confirmed these findings at the protein level, showing increased STC1 expression following IL‐1β stimulation and reduced expression after ART treatment (Figure 4E,F). Immunofluorescence staining in primary chondrocytes showed a similar pattern (Figure 4G,H).

We next performed immunofluorescence staining on OA patient samples to further assess STC1 expression in human cartilage. STC1 fluorescence intensity was higher in medial tibial plateau cartilage than in paired lateral tibial plateau cartilage from the same patients, which was further supported by quantitative analysis (Figure 4I,J).

In addition, immunofluorescence staining in mouse articular cartilage showed increased STC1 signals in the DMM + Vehicle group and reduced signals in ART‐treated mice (Figure 4K,L).

Single‐gene GSEA of STC1 showed enrichment of gene sets associated with inflammatory response, immune regulation, and ECM‐related processes (Figure 4M).

These results indicate that STC1 expression is altered under OA‐related conditions and is associated with ART treatment. Computational structural analysis suggested potential structural compatibility between ART and STC1 (Figure S3A,B); however, these analyses were not intended to establish direct binding interactions. The reduction in STC1 mRNA following ART treatment suggests that transcriptional regulation contributes to the observed decrease in STC1 expression.

### 3.5 Artemotil Treatment Is Associated With Altered SMAD2/3 Phosphorylation in Osteoarthritic Chondrocytes

**FIGURE 5:**
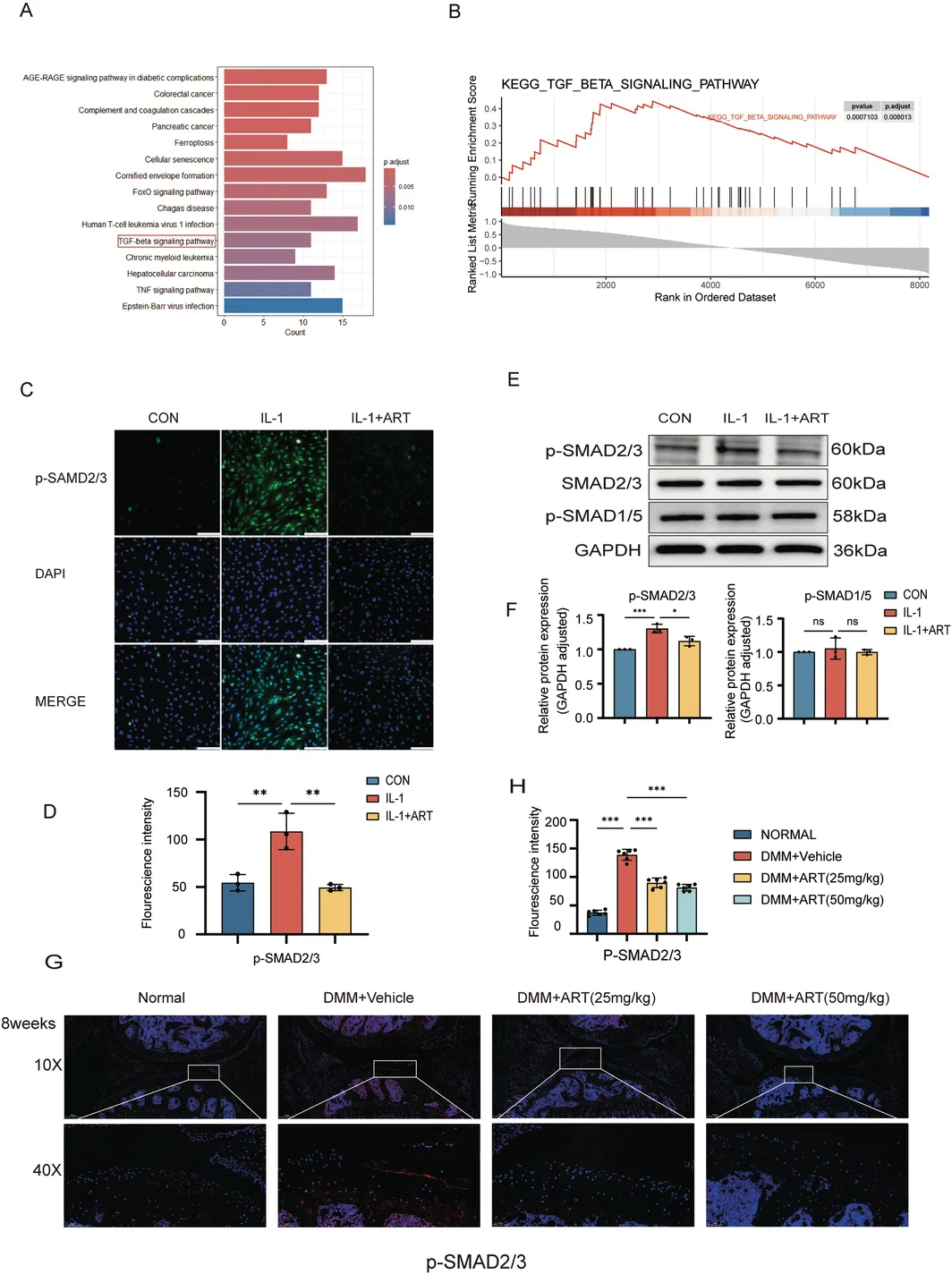
Artemotil treatment is associated with altered SMAD2/3 phosphorylation in osteoarthritic chondrocytes (A) KEGG pathway enrichment analysis of differentially expressed proteins derived from proteomic data. (B) Single‐gene GSEA of STC1 showing enrichment of TGF‐β–related signalling pathways. (C, D) Immunofluorescence staining and quantification of p‐SMAD2/3 in primary chondrocytes. Scale bar, 100 μm. (E, F) Western blotting and quantification of p‐SMAD2/3, total SMAD2/3, and p‐SMAD1/5 in primary chondrocytes. (G, H) Immunofluorescence staining and quantification of p‐SMAD2/3 in articular cartilage. Data are presented as the mean ± SD, with individual data points shown. In vitro experiments were performed in triplicate (*n* = 3), and in vivo experiments included 6 mice per group. Statistical analysis was performed using one‐way ANOVA followed by Bonferroni post hoc test. *p < 0.05, **p < 0.01, ***p < 0.001.

Quantitative proteomic analysis was used to explore pathways associated with ART treatment in OA chondrocytes. KEGG enrichment analysis showed that differentially expressed proteins were associated with multiple pathways, including inflammation‐related and signal transduction pathways, such as the AGE–RAGE signalling pathway, TNF signalling pathway, complement and coagulation cascades, and the TGF‐β signalling pathway (Figure 5A).

Single‐gene GSEA of STC1 showed enrichment of gene sets associated with TGF‐β–related signalling pathways (Figure 5B).

Immunofluorescence staining showed increased nuclear p‐SMAD2/3 signals following IL‐1β stimulation and lower signals following ART treatment (Figure 5C,D). Western blotting further showed increased C‐terminal SMAD2/3 phosphorylation in IL‐1β‐treated chondrocytes and lower levels following ART treatment, whereas total SMAD2/3 levels remained relatively unchanged (Figure 5E,F). No statistically significant group‐dependent difference in p‐SMAD1/5 phosphorylation was detected in primary chondrocytes.

To further assess these signalling changes in vivo, p‐SMAD2/3 was examined in articular cartilage. Higher p‐SMAD2/3 staining was observed in the DMM + Vehicle group compared with the Control group, whereas lower staining was observed in ART‐treated mice (Figure 5G,H). Additional immunohistochemical assessment of p‐SMAD1/5 did not reveal a statistically significant group‐dependent difference among the Control, DMM + Vehicle, and ART‐treated groups (Figure S3C,D).

These findings demonstrate increased SMAD2/3 phosphorylation under the OA‐related experimental conditions examined, whereas no detectable group‐dependent alteration in SMAD1/5 phosphorylation was observed.

### 3.6 STC1 Overexpression Enhances Fibrosis‐Associated Phenotypic Remodelling in Chondrocytic Cells

**FIGURE 6:**
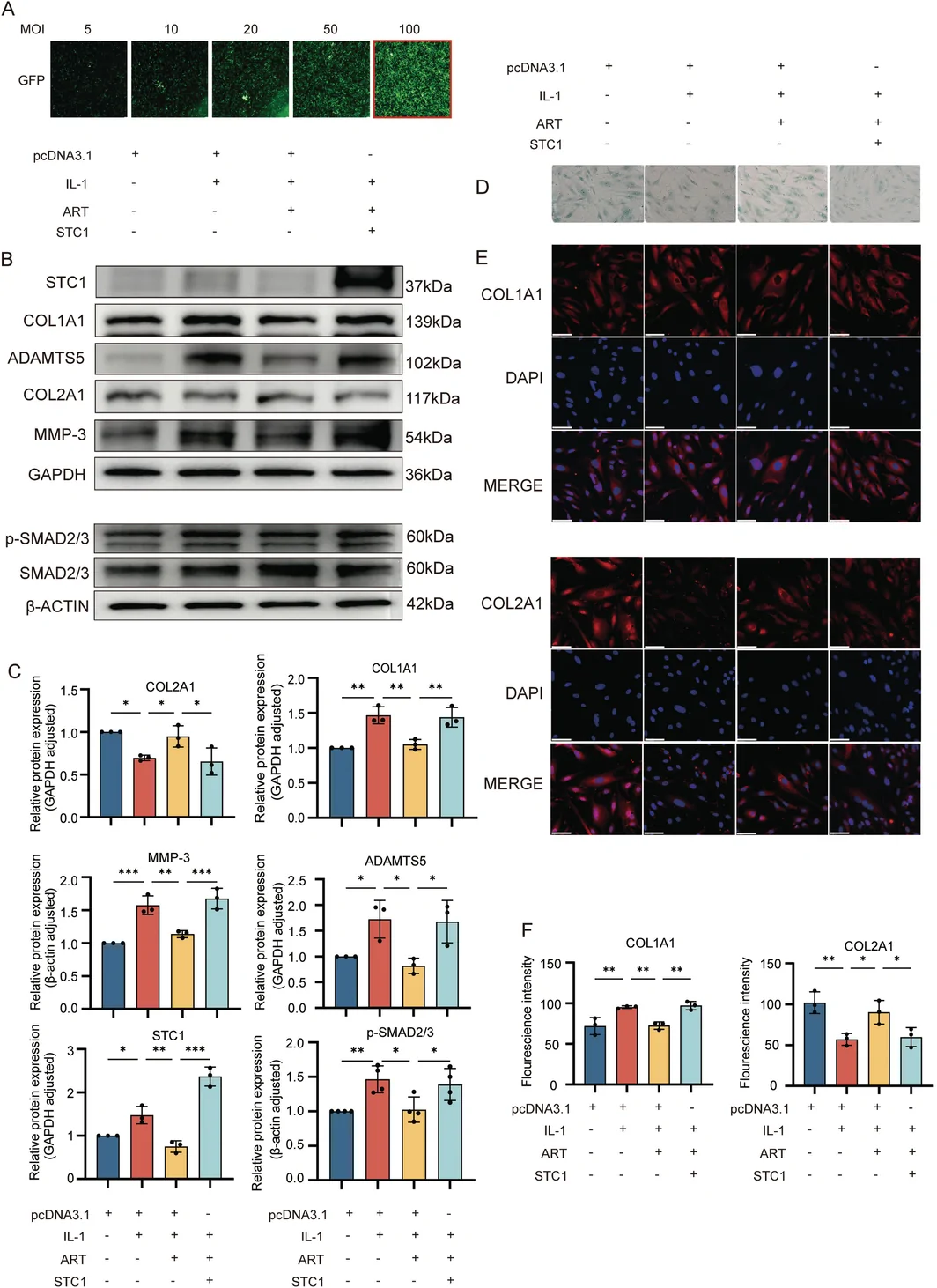
STC1 overexpression enhances fibrosis‐associated phenotypic remodelling and alters Artemotil responsiveness. (A) Fluorescence imaging of SW1353 cells transduced with ZsGreen‐labelled lentiviral vectors at different MOIs. (B, C) Western blotting and quantification of STC1, COL1A1, COL2A1, ADAMTS5, MMP‐3, and p‐SMAD2/3 in different treatment groups. (D) Alcian blue staining showing extracellular matrix deposition. (E, F) Immunofluorescence staining and quantification of COL1A1 and COL2A1. Scale bar, 25 μm. Data are presented as the mean ± SD, with individual data points shown (*n* = 3). Statistical analysis was performed using one‐way ANOVA followed by Bonferroni post hoc test. *p < 0.05, **p < 0.01, ***p < 0.001.

To further investigate the role of STC1 in ART‐associated fibrotic phenotypic alterations, we established an STC1‐overexpression model in SW1353 chondrosarcoma‐derived chondrocytic cells. The multiplicity of infection (MOI) for lentiviral transduction was evaluated based on ZsGreen fluorescence, and an MOI of 100 was selected for subsequent experiments (Figure 6A).

Western blotting showed that IL‐1β stimulation increased the expression of STC1, COL1A1, ADAMTS5, and MMP‐3, accompanied by increased p‐SMAD2/3 levels, whereas COL2A1 expression was reduced compared with that in the control group (Figure 6B,C). ART treatment reduced STC1 expression, decreased levels of COL1A1, ADAMTS5, and MMP‐3, increased COL2A1 expression, and reduced p‐SMAD2/3 levels.

Following STC1 overexpression in the presence of ART, increased STC1 expression was observed, accompanied by increased COL1A1, ADAMTS5, and MMP‐3 levels, reduced COL2A1 expression, and increased p‐SMAD2/3 levels compared with those in the ART‐treated group (Figure 6B,C).

Immunofluorescence staining showed low COL1A1 and high COL2A1 signals in the control group. IL‐1β stimulation increased COL1A1 and reduced COL2A1 signals, whereas ART treatment partially reversed these changes. STC1 overexpression in the presence of ART was associated with increased COL1A1 and reduced COL2A1 signals compared with those in the ART‐treated group (Figure 6E,F). Similar trends were observed with Alcian blue staining (Figure 6D).

These results support an association between STC1 expression and fibrosis‐associated phenotypic remodelling and suggest that STC1 expression may influence the responsiveness of chondrocytes to Artemotil treatment.

### 3.7 Functional Association Between STC1 and TGF‐β/SMAD2/3 Signalling During Chondrocyte Fibrotic Remodelling

**FIGURE 7:**
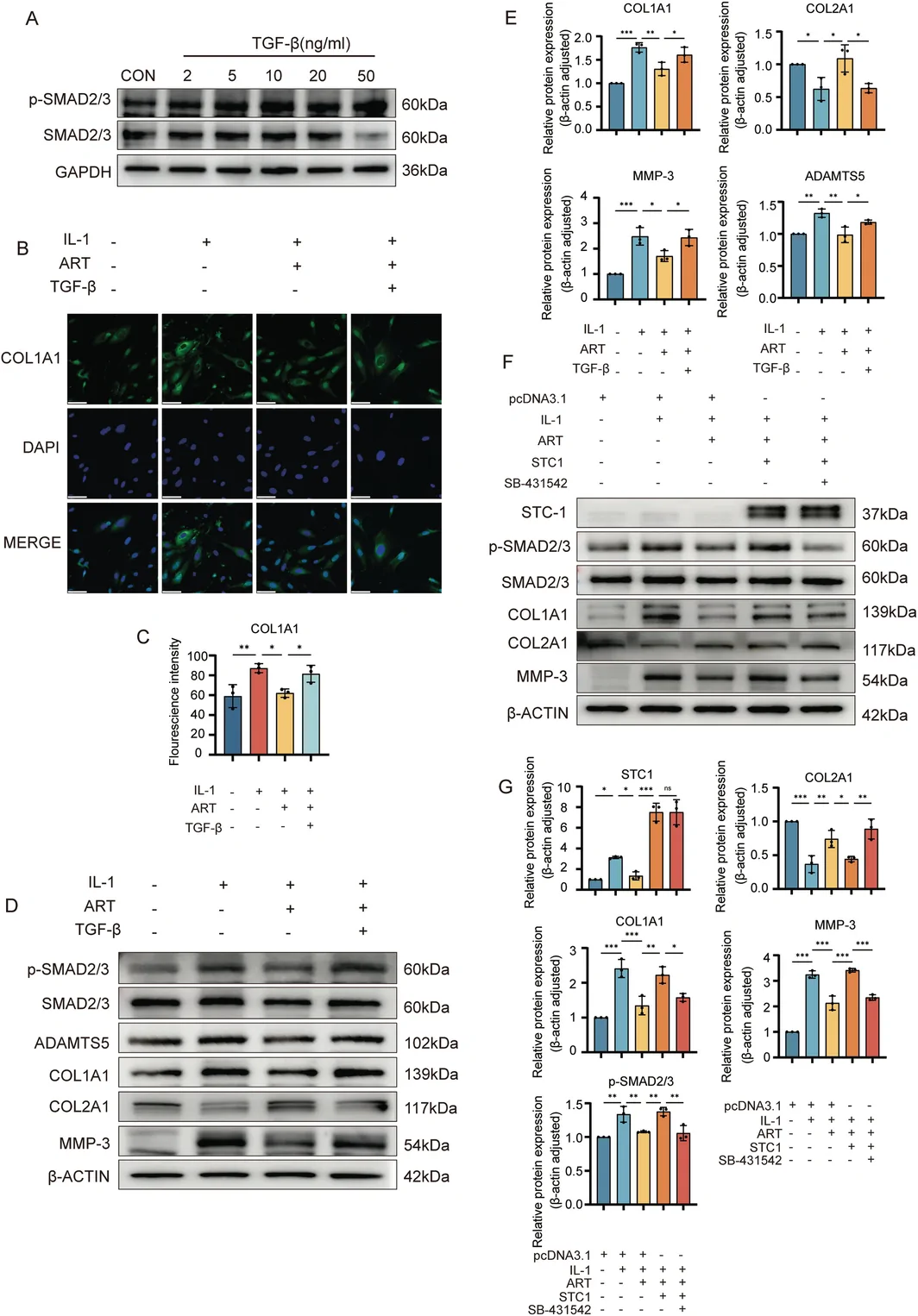
Functional association between STC1 and TGF‐β/SMAD2/3 signalling during chondrocyte fibrotic remodelling (A) Western blotting of p‐SMAD2/3 in SW1353 cells treated with increasing concentrations of TGF‐β. (B–E) Western blotting and immunofluorescence analyses of p‐SMAD2/3 and fibrosis‐related markers in SW1353 cells treated with IL‐1β, Artemotil, and TGF‐β. (F, G) Western blotting and quantification of p‐SMAD2/3 and fibrosis‐related markers in STC1‐overexpressing SW1353 cells treated with the TGF‐β receptor inhibitor SB‐431542. Data are presented as the mean ± SD, with individual data points shown (*n* = 3). Statistical analysis was performed using one‐way ANOVA followed by Bonferroni post hoc test. *p < 0.05, **p < 0.01, ***p < 0.001.

To examine the effects of TGF‐β on SMAD2/3 phosphorylation, we treated SW1353 cells with increasing concentrations of TGF‐β. Western blotting demonstrated that p‐SMAD2/3 levels increased with increasing TGF‐β concentrations, whereas total SMAD2/3 levels remained unchanged (Figure 7A).

To further assess the effects of TGF‐β on ART‐associated changes, we treated SW1353 cells with IL‐1β and TGF‐β with or without ART. Western blotting and immunofluorescence analyses showed that IL‐1β increased p‐SMAD2/3, COL1A1, ADAMTS5, and MMP‐3 levels and reduced COL2A1 expression (Figure 7B–E). These changes were attenuated in the ART‐treated group. In the presence of both ART and TGF‐β, increased p‐SMAD2/3, COL1A1, ADAMTS5, and MMP‐3 levels and reduced COL2A1 levels were observed compared with those observed with ART treatment alone.

To further examine the relationship between STC1 and TGF‐β signalling, STC1‐overexpressing SW1353 cells were treated with the TGF‐β receptor inhibitor SB‐431542. Compared with the IL‐1β + ART group, STC1 overexpression increased p‐SMAD2/3, COL1A1, and MMP‐3 levels and reduced COL2A1 expression (Figure 7F,G). Treatment with SB‐431542 was associated with reduced p‐SMAD2/3, COL1A1, and MMP‐3 levels and increased COL2A1 expression.

These findings support a functional association between STC1 expression, TGF‐β‐responsive signalling, and SMAD2/3 phosphorylation under the experimental conditions examined.

## 4 Discussion

Cartilage fibrosis has emerged as an important pathological component of OA progression and is characterized by a shift from hyaline cartilage toward a fibrocartilage‐like phenotype [32]. In addition to extracellular matrix degradation, the transition from hyaline cartilage toward a fibrocartilage‐like phenotype contributes to alterations in tissue composition and mechanical function [7, 33]. The present study identifies STC1‐associated TGF‐β/SMAD2/3 signalling as a molecular event linked to fibrosis‐associated chondrocyte remodelling in OA. STC1 expression was elevated in IL‐1β‐stimulated chondrocytes, experimental OA cartilage, and human OA cartilage. In the experimental OA models, increased STC1 expression was accompanied by increased SMAD2/3 phosphorylation and fibrosis‐related marker changes. Artemotil treatment reduced STC1 expression and SMAD2/3 phosphorylation and attenuated fibrotic and catabolic alterations in chondrocytes and DMM‐induced OA cartilage.

Previous studies have suggested that cartilage fibrosis contributes to osteoarthritis progression [34, 35]. However, the molecular regulators governing this process remain incompletely understood. In contrast, the present findings highlight fibrotic phenotypic alterations, reflected by increased COL1A1 and decreased COL2A1 under inflammatory conditions. Consistent with the in vitro findings, reduced COL2A1 staining was also observed in DMM articular cartilage, whereas higher COL2A1 signals were detected following ART treatment. Together with the alterations in COL1A1, these findings support fibrosis‐related remodelling characterized by changes in hyaline cartilage‐associated matrix composition. These alterations may indicate a shift from hyaline cartilage toward a fibrocartilage‐like phenotype, which has been linked to changes in tissue composition and mechanical properties. The observed effects of ART on these markers extend current understanding beyond classical catabolic processes and suggest that modulation of fibrosis‐associated remodelling may contribute to its observed chondroprotective effects in osteoarthritis. The current findings further link Artemotil treatment to altered STC1 expression and STC1‐associated signalling activity.

Proteomic analysis identified STC1 as a differentially expressed protein associated with ART treatment. This observation was consistently validated across multiple experimental systems. STC1 has been reported to participate in cellular stress responses, mitochondrial regulation, and tissue remodelling in different biological contexts [22, 36]. Previous studies have described variable STC1 expression patterns in OA‐related tissues, with differences reported between cartilage and synovium, suggesting context‐dependent regulation [23, 37]. The present findings extend previous observations by suggesting that STC1 is associated with chondrocyte fibrotic remodelling. Increased STC1 expression was consistently accompanied by elevated COL1A1 expression, reduced COL2A1 expression, and increased SMAD2/3 phosphorylation, although causality remains to be further established. These observations support a possible functional contribution of STC1 to fibrosis‐associated phenotypic changes in osteoarthritic cartilage. Although proteomic analysis was performed in a cell‐based system, the expression pattern of STC1 was consistently validated across primary chondrocytes, mouse cartilage, and human cartilage samples.

One notable finding of this study is the association between STC1 expression and TGF‐β/SMAD2/3 signalling activity. Although the precise molecular relationship remains to be established, STC1 overexpression was accompanied by increased SMAD2/3 phosphorylation and enhanced expression of fibrosis‐related markers. Pharmacological inhibition of TGF‐β signalling partially attenuated these effects, supporting a functional association between STC1 and TGF‐β/SMAD2/3 signalling during chondrocyte remodelling.

Rather than functioning as a simple downstream effector, STC1 may participate in a broader fibrosis‐associated signalling network under inflammatory conditions. Previous studies have linked STC1 to tissue remodelling, mitochondrial stress responses, and TGF‐β‐related pathways in multiple organs [38]. The present findings extend these observations to osteoarthritic cartilage and support a potential association between STC1 expression and cartilage fibrosis.

TGF‐β/SMAD signalling plays complex and context‐dependent roles in articular cartilage [39, 40]. Canonical ALK5–SMAD2/3 signalling has been associated with maintenance of cartilage matrix homeostasis, whereas a relative shift toward ALK1–SMAD1/5 signalling has been linked to chondrocyte hypertrophy and catabolic responses [41]. Blaney Davidson et al. demonstrated that aging and osteoarthritic cartilage exhibit an increased ALK1/ALK5 ratio, with ALK1 expression associated with MMP‐13 and ALK5 expression associated with aggrecan and COL2A1 [42]. More recent studies have emphasized that TGF‐β signalling within the joint is tissue‐specific, non‐linear, and highly context‐dependent [43], with its biological output further influenced by receptor usage and downstream SMAD selection [44].

The increased C‐terminal SMAD2/3 phosphorylation observed in the present study differs from reports describing attenuation of the homeostatic ALK5–SMAD2/3 branch during OA. However, increased p‐SMAD2/3 has also been reported in other OA‐related settings. Xu et al. detected increased TGF‐β1 and p‐SMAD2/3 in articular chondrocytes from mouse OA models, whereas p‐SMAD1 was only weakly detectable [45]. Deroyer et al. further showed that TGF‐β‐associated p‐SMAD2/3 signalling participates in a fibrosis‐like phenotypic transition of osteoarthritic chondrocytes [46]. These apparently divergent observations indicate that the direction and biological consequence of SMAD2/3 signalling cannot be inferred solely from the presence of OA and may depend on cellular state and local signalling context.

An additional consideration is that C‐terminal SMAD2/3 phosphorylation alone does not provide a complete assessment of downstream pathway function. Consistent with this complexity, Ge et al. showed that increased p‐SMAD2 following PPM1A deletion was associated with protection against DMM‐induced cartilage degeneration, further indicating that the biological consequence of SMAD2 phosphorylation depends on signalling context rather than phosphorylation level alone [47]. OA‐related inflammatory signalling can modify the SMAD2/3 linker region and alter the transcriptional consequences of TGF‐β signalling. Thielen et al. demonstrated that IL‐1β‐associated inflammation interferes with the protective effects of TGF‐β through regulation of SMAD2/3 linker phosphorylation [48]. This point is particularly relevant to the present in vitro model, in which chondrocytes were exposed to IL‐1β. Because we measured C‐terminal SMAD2/3 phosphorylation but did not assess linker phosphorylation, ALK1/ALK5 receptor balance, or receptor‐specific downstream transcriptional activity, our p‐SMAD2/3 results should be interpreted as a biochemical signalling readout rather than as evidence that canonical ALK5–SMAD2/3 signalling is globally activated or uniformly detrimental in OA.

Our observations regarding SMAD1/5 also differ from previous reports. Jaswal et al. demonstrated activation of BMP signalling, reflected by increased p‐SMAD1/5, in experimental OA and further showed that suppression of BMP signalling attenuated OA progression [49]. In contrast, we did not detect a statistically significant group‐dependent alteration in p‐SMAD1/5 in either cultured chondrocytes or articular cartilage. However, SMAD1/5 activation has not been uniformly observed across all OA studies; Xu et al. reported increased p‐SMAD2/3 in mouse OA cartilage while p‐SMAD1 was only weakly detectable [45]. The basis for these differences cannot be determined from the present data. Variations in local ligand and receptor availability, cartilage region, inflammatory status, phosphorylation dynamics, disease stage, experimental model, and analytical sensitivity may contribute. Importantly, the absence of a detectable p‐SMAD1/5 difference in the present study does not exclude a role for BMP–SMAD1/5 signalling in OA. Rather, our data indicate that no measurable group‐dependent alteration was detected under the specific experimental conditions examined.

The experimental design combined IL‐1β‐induced inflammatory models with a DMM‐induced OA model, providing complementary information at the cellular and tissue levels. IL‐1β stimulation induces reproducible catabolic responses in chondrocytes [31], whereas the DMM model reflects progressive structural alterations observed in OA [50]. The consistency between in vitro and in vivo findings supports the association between ART treatment and modulation of cartilage remodelling processes. In addition, although ART was administered intraperitoneally in this study, intra‐articular delivery may be a more clinically relevant approach to enhance local drug retention while minimizing systemic exposure, which warrants further investigation. Future studies evaluating local delivery strategies and pharmacokinetic profiles will be required to assess clinical feasibility.

Notably, the present study integrates proteomic screening, experimental osteoarthritis models, and human cartilage validation to identify STC1 as a candidate regulator associated with chondrocyte fibrotic remodelling. Although STC1 has previously been implicated in tissue remodelling and fibrosis in other organs, evidence linking STC1 to cartilage fibrosis remains limited. The current findings provide preliminary evidence supporting a role for STC1‐associated signalling in osteoarthritis‐related cartilage remodelling.

Several limitations should be considered when interpreting the present findings. First, the proteomic analysis was performed in a cell‐based system, and the functional investigation of STC1 was limited to gain‐of‐function experiments. Additional loss‐of‐function studies will be required to further define the causal contribution of STC1 to osteoarthritis‐associated fibrotic remodelling. Moreover, although primary chondrocytes were used for expression validation, STC1 overexpression and pathway‐modulation experiments were performed in SW1353 chondrosarcoma‐derived chondrocytic cells. These findings therefore require further validation in primary chondrocytes and, ultimately, in vivo models with targeted modulation of STC1. Although SMAD2/3 and SMAD1/5 phosphorylation were assessed, the present study did not directly determine ALK1/ALK5 receptor balance, SMAD2/3 linker phosphorylation, or receptor‐specific downstream transcriptional responses. Therefore, the phosphorylation measurements should not be interpreted as a comprehensive assessment of TGF‐β/BMP pathway activity.

The mechanisms underlying ART‐mediated regulation of STC1 expression also remain incompletely defined. In particular, direct biochemical evidence for a physical interaction between Artemotil and STC1 is currently unavailable and will require validation using dedicated biophysical approaches. Systemic safety assessment was limited because longitudinal body‐weight changes were not systematically analysed as a safety outcome, and kidney histology and serum biochemical parameters were not evaluated.

The present study primarily characterized fibrosis‐related and hyaline cartilage phenotypes using COL1A1 and COL2A1, respectively. Hypertrophic markers such as COL X were not systematically assessed. Accordingly, the current data do not provide a comprehensive evaluation of chondrocyte hypertrophic differentiation or cartilage mineralization at the molecular level.

Limitations related to the animal experimental design should also be acknowledged. Male and female mice were pooled for the primary analyses. Biological sex can influence OA development and associated pathological responses, and sex‐dependent differences in cartilage degeneration and molecular phenotypes have been reported in experimental OA models [51, 52]. Although both sexes were included and distributed across the experimental groups to reduce potential allocation bias, the present study was not adequately powered to assess sex‐specific effects or sex‐by‐treatment interactions. Potential differences in the response to Artemotil between male and female mice therefore cannot be excluded and should be examined in appropriately powered sex‐stratified studies.

Finally, the sample size for the animal experiments was not determined using a formal a priori power analysis. Although the group size used in the present study was comparable to that employed in a recent DMM‐based OA study [53], this does not substitute for a prospectively defined sample‐size calculation. The statistical power of individual in vivo endpoints therefore cannot be established prospectively from the present design. Future confirmatory studies should define a prespecified primary outcome and determine the required sample size according to the expected effect size, variability, significance level, and desired statistical power.

In summary, the present study supports a functional association between STC1, TGF‐β‐responsive SMAD2/3 signalling, and fibrosis‐associated chondrocyte remodelling in osteoarthritis. Artemotil treatment was associated with reduced STC1 expression and SMAD2/3 phosphorylation and attenuation of fibrosis‐related phenotypic changes. These findings support further investigation of STC1‐associated mechanisms and fibrosis‐related remodelling pathways in osteoarthritis progression.

## 5 Conclusion

The present study provides evidence that STC1 is associated with fibrosis‐related chondrocyte remodelling and altered SMAD2/3 phosphorylation under OA‐related experimental conditions. Increased STC1 expression was consistently associated with fibrosis‐related phenotypic alterations and increased SMAD2/3 phosphorylation across the experimental models examined, while altered STC1 expression was independently validated in human OA cartilage. Artemotil treatment attenuated these changes. These findings identify STC1 as a candidate regulator of OA‐related cartilage remodelling and support further investigation of the receptor‐specific and context‐dependent mechanisms underlying STC1‐associated signalling.

## Author Contributions

**Guang Zhu:** writing – original draft, conceptualization, investigation, methodology. **Yang Yang:** investigation, validation, software. **Ye Ma:** investigation, software, data curation. **Gang Wu:** resources, formal analysis. **Kuanmin Tian:** resources, validation. **Xiaoxin He:** methodology, validation. **Rui Wang:** funding acquisition, validation. **Penggang Ma:** validation, formal analysis, resources. **Zhiqun Tang:** resources. **Qunhua Jin:** writing – review and editing, funding acquisition, formal analysis, project administration, supervision.

## Funding

This study was supported by the National Natural Science Foundation of China (Nos. 82160433, U22A20285), the Key R&D Project of Autonomous Region (No. 2023BEG02018), the Autonomous Region Major Scientific and Technological Achievements Transformation Project (No. 2023CJE09037), and Ningxia Medical University General Hospital “Medical Engineering Special” (No. NYZYYG‐001).

## Ethics Statement

All animal procedures in this study were conducted in strict accordance with the Guidelines for the Care and Use of Laboratory Animals established by the National Institutes of Health. The experimental protocol was reviewed and approved by the Ningxia Medical University Institutional Animal Care and Use Committee (IACUC‐H2025072). C57BL/6J mice were housed under specific pathogen–free conditions, and all efforts were made to minimize animal suffering throughout the study.

Human cartilage samples were used in this study. The study protocol was approved by the Medical Ethics Review Committee of General Hospital of Ningxia Medical University (Approval No. KYLL‐2024‐153), and written informed consent was obtained from all participants in accordance with institutional guidelines. The use of commercially available human chondrocyte cell lines complied with relevant institutional and national regulations.

## Consent

No individual person's data, images, or identifiable information are included in this study. Therefore, consent for publication is not applicable.

## Conflicts of Interest

The authors declare that they have no competing financial or personal interests that could have influenced the work reported in this manuscript. No author holds any financial stake, advisory role, patent application, or other conflict related to the study's design, data collection, analysis, or interpretation.

## Supporting information


**Figure S1:** Characterization of primary mouse articular chondrocytes and additional validation of cartilage phenotype. (A) Representative double immunofluorescence staining of SOX9 and COL2A1 in primary mouse articular chondrocytes. SOX9 is shown in green, COL2A1 in red, and nuclei were counterstained with DAPI. (B) Quantification of SOX9/COL2A1 double‐positive cells among total DAPI‐positive cells. (C) RT‐qPCR analysis of COL1A1 and MMP‐3 mRNA expression in control, IL‐1β, and IL‐1β + Artemotil groups. (D) Representative COL2A1 immunofluorescence staining in articular cartilage from the Control, DMM + Vehicle, DMM + Artemotil (25 mg/kg), and DMM + Artemotil (50 mg/kg) groups at 8 weeks. (E) Quantification of COL2A1 fluorescence intensity in articular cartilage. Quantitative data are presented as the mean ± SD with individual data points shown. For chondrocyte characterization, three independent cell isolations were analysed (*n* = 3). RT–qPCR experiments were performed with three independent biological replicates (*n* = 3). In vivo COL2A1 analysis included six mice per group (*n* = 6). *p < 0.05, **p < 0.01, ***p < 0.001.

**Figure S2:** Histological assessment of liver tissues following Artemotil treatment. Representative H&E staining of liver sections from the Control, DMM + Vehicle, DMM + Artemotil (25 mg/kg), and DMM + Artemotil (50 mg/kg) groups. No obvious histopathological abnormalities were observed under the experimental conditions examined. Scale bar, 50 μm.

**Figure S3:** Computational structural analysis of Artemotil–STC1 compatibility and assessment of p‐SMAD1/5 in articular cartilage. (A) Molecular docking analysis showing the predicted structural compatibility between Artemotil and STC1. (B) Molecular dynamics analyses of the predicted Artemotil–STC1 complex, including RMSD, radius of gyration (Rg), hydrogen‐bond number, solvent‐accessible surface area (SASA), and RMSF. These computational analyses represent theoretical structural predictions and were not intended to establish direct biochemical binding. (C) Representative immunohistochemical staining of p‐SMAD1/5 in articular cartilage from the Control, DMM + Vehicle, and DMM + Artemotil (25 mg/kg) groups. (D) Quantitative analysis of the p‐SMAD1/5‐positive area in articular cartilage. Data are presented as the mean ± SD with individual data points shown (*n* = 6 per group). ns, not significant.

**Table S1:** Primer sequences used for mouse RT–qPCR.

## Data Availability

The mass spectrometry proteomics data have been deposited to the ProteomeXchange Consortium via the iProX partner repository with the dataset identifier PXD077828. The dataset is currently under private access for peer review and will be made publicly available upon publication. The datasets generated and analysed during this study are available from the corresponding author on reasonable request. All data supporting the findings of this study are included in the article and its Supporting Information.
