# Supplementary material for: Artemotil Attenuates Fibrosis‐Associated Chondrocyte Remodelling in Osteoarthritis in Association With STC1‐Related TGF‐β/SMAD2/3 Signalling
Source: J Cell Mol Med. 2026 Sep 16;30(18):e71374. doi: 10.1111/jcmm.71374 (PMC13580662; doi:10.1111/jcmm.71374)
Supplement: Supplementary file 1 [file JCMM-30-e71374-s003.docx]

| Gene | Primer | Sequence (5′→3′) |
| --- | --- | --- |
| STC1 | STC1-F | AAGCCTGCTGGAATGTGATG |
|  | STC1-R | GGTGCGTCTCCTGTTGAAG |
| COL1A1 | COL1A1-F | GGTATTGCTGGTGCTCCTG |
|  | COL1A1-R | CTCGCTTTCCTTCCTCTCC |
| MMP3 | MMP3-F | CCACTCTATCACTCACTCACAG |
|  | MMP3-R | GACAGCATCAAAGGACAAAGC |
| GAPDH | GAPDH-F | GGAGCGAGATCCCTCCAAAAT |
|  | GAPDH-R | GGCTGTTGTCATACTTCTCATGG |

Table S1. Primer sequences used for mouse RT–qPCR.
